# Challenges and motivators to physical activity faced by retired men when ageing: a qualitative study

**DOI:** 10.1186/s12889-018-5517-3

**Published:** 2018-05-15

**Authors:** Ebba Langum Bredland, Sylvia Söderström, Kjersti Vik

**Affiliations:** Faculty of Medicine & Health Science, Department of Neuromedicine and Movement Science, Norwegian University of Medicine & Health Sciences, Trondheim, Norway

**Keywords:** Active ageing, Perceived challenges, Motivators, Social influence, Elderly men, Physical activity, Qualitative study

## Abstract

**Background:**

Active ageing reflects the message from World Health Organisation about addressing the challenge faced by the rapidly ageing population. Knowledge about barriers and facilitators to an active lifestyle must be given more attention. In addition, men seem to participate less in cultural activities and less in fall-prevention groups than women do. When mostly women work with the elderly in primary care, one might question whether the activities offered to older men meet their activity preferences. The aim of this study is to provide new knowledge about challenges and motivators encountered by retired men in maintaining physical activity when ageing.

**Methods:**

Nine retired men, aged between 66 and 83, wrote a Time Geographic Diary for 7 days each. Two focus group discussions with the men were held. A Systemic Text Condensation was used to analyse the data.

**Results:**

The analyses identified three categories to describe challenges in being physical active: differences between men and women; meaningful physical activity; and environmental - especially socio-cultural - constraints. Motivating conditions were seen as: new activities to get younger friends, and more information about how to cope.

**Conclusions:**

To achieve the aim of active ageing, service providers as well as local authorities need to have a better understanding of the challenges retired men encounter when ageing. This study highlights vital aspects of the challenges faced by retired men in maintaining their physical activity level.

## Background

In western society, there is a growing concern about inactivity having a negative impact on health in the population. Both policy and research during the past few decades have focused on exercise, and there are recommendations for the number of minutes one should exercise per week [1]. Lately, the emphasis has increasingly been on how a sedentary lifestyle is a challenge to good health [2–5]. Consequently, understanding the barriers to physical activity and how to facilitate a more active everyday lifestyle for the population, must be given more attention [6].

Active ageing reflects the message from World Health Organisation [7] about addressing the challenge faced by the rapidly ageing population. Sedentary change in the population as a whole is also a challenge for seniors. From focusing on exercising in the gym, more emphasis is now being put on the positive effect of outdoor everyday activities for seniors, like gardening [8, 9] and walking [10, 11]. Outdoor walking especially has been given a lot of attention in the past few years. However, the various characteristics of each individual’s physical environment will influence this activity [11–13]. According to Tsai et al. [14], walking in the community between different life-space levels is important in promoting physical activity. Because walking outdoors is recommended for seniors, there is a need to explore how barriers, motivators and environmental factors can facilitate walking and spending time outdoors for the elderly [11, 15]. Better information is needed to be able to take responsibility for one’s own health, and this also implies of one’s physical activity.

It would be helpful for each municipality to map the environment in order to make an overview of potential barriers to physical activity for the population. Haselwandter et al. [12] looked at the built environment and pointed out that the individual’s physical environment can either encourage or be a barrier to physical activity. Jefferis et al. [16] write about falling as a barrier to physical activity in community-dwelling older men. Fear of falling is associated with restriction of activities, even more than if the person has actual had experience of falling [17]. In addition, advice about fall prevention can be perceived as a threat to the older people’s identity and autonomy, and potentially patronizing and distressing [18].

Experience from the field of practice and evaluations from primary care reports in a Norwegian municipality, point to differences between men and women when it comes to participation in the municipality’s activity programmes. The seniors appear to be quite active in their everyday life, even though they suffer from many medical conditions [19]. Nevertheless, men seem to participate less in cultural activities and less in fall-prevention groups [20]. It is mostly women who work with the elderly in primary care, and one might question whether the activities offered to older adults meet the activity preferences of older men. There is a marked gender difference [21] and therefore a need to provide a deeper insight into the challenges faced by men in terms of keeping up their physical activities when ageing. For examples, what are the factors which may influence men’s participation in outdoor walking, what do they perceive as obstacles, and what keeps them from being physically active when ageing? How can retired men’s experience provide useful advice to health professions and other men when they meet obstacles to being physically active?

The aim of this pilot study is therefore to explore and describe challenges and motivators to physical activity faced by retired men when ageing.

## Methods

To gather information about the challenges to continue physical activity in their older years perceived by retired men, the combined method of Time Geographic Diary and Focus Group Discussions was chosen.

Time Geographic Diary (TGD) provides a tool for systematic studies of activities of everyday life [22]. The time-geographic perspective focuses on what people do, and how everyday life is shaped by what the individual gives preference to and decides to do. The participants wrote down the time they started any new activity, what the activity was, where it took place and who they did it with, and they were free to make comments. In this study, they wrote TGD for 1 week each, the total data consisted of information from 63 days. This method was chosen both as a data gathering method and a method to raise awareness to give them an impression of their own activities.

The Focus Group Discussion (FGD) method was chosen with the intention of encouraging the participants to explore and discuss their experiences of how to continue to be physically active after retiring. This method is suitable for gaining an understanding of how the participants make sense of and discuss topics of interest. Furthermore, being among others who share many of the same experiences in a permissive/inclusive environment can facilitate participants to express both positive and negative opinions more easily [23].

The study was approved by the Norwegian Social Science Data Services.

### Participants

The study took place in a Norwegian town where the local authority is responsible for organising home care services and health promotion programmes. The participants were recruited from the “Infocentre for seniors”, an information office for health promotion among older adults. The staff at the centre was given information about the study and asked to recruit participants. A purposeful sampling strategy were chosen [24] and the inclusion criteria were: retired men, no home care services, different activity levels and capable and interested in diary writing for 1 week. Twelve men were asked to participate, however after an initial talk with each man, three men decided not to participate. Thus, nine men were included in the study. In line with purposeful sampling strategy [24], the men differed in age and education, type of housing and living arrangements (alone or with spouse). In addition men from different activity groups [25] were included, see Table 1. Physical activity can be divided into unstructured activity incorporated in daily life – so called Non-exercise physical activity NEPA [2, 3], and exercising. The four groups were [25]; 1. Exercising and doing NEPA, 2. Exercising – no NEPA, 3. NEPA – no exercising 4. No NEPA - no exercising. Their age ranged in age from 66 to 83. None of the men knew each other from previous meetings. Before the start, the participants were given written information about the study, and signed an informed consent statement.

**Table 1:** Participant Characteristics

	Age	Living with	Comments	Housing	Activity group [25]
A.	83	Alone	Wife in nursing home	Terraced house	Gr. 1 Exercising/NEPA^a^
B.	82	With wife	Wife retired	Apartment	Gr. 2 Exercising/No NEPA
C.	66	Alone	Wife in nursing home	Apartment	Gr. 3 NEPA/ No exercising
D.	80	Alone	Wife deceased	Apartment	Gr. 3 NEPA/ No exercising
E.	81	Alone	Divorced	Semi-detached house	Gr. 3 NEPA/ No exercising
F.	79	With wife	Wife still working	House	Gr. 1 Exercising/NEPA
G.	71	With wife	Wife retired	House	Gr. 1 Exercising/NEPA
H.	73	With wife	Wife with dementia at home	House	Gr. 3 NEPA/No exercising
I.	73	With wife	Wife still working	Apartment	Gr. 4 No Exercising/No NEPA

^a^*NEPA* Non-Exercise Physical Activity

### Data collection

Nine participants wrote a TGD for 1 week each. The first author met them before they started writing in order to give information, encourage them to write comments and answer any questions. This author also meet them after the diaries had been written in order to ensure the week was an ordinary week, and to talk about their experience and any additional comments. Meeting each participant twice seemed to make them understand the importance of this study, and perhaps made them motivated to make more comments during the week.

Studies have shown that a group size between four and eight enables everybody to participate freely in FGD [24, 26]. Two discussions took place at a meeting room at the Infocentre in the municipality. This is a neutral meeting room in the city centre. All nine men who had written a diary were invited; six of them attended. Two of them were unable to come because they lived abroad in the winter, and one was unwell. A semi-structured interview guide with open-ended questions had been sent to the participants some days before the meeting. The discussion centred on “Retired men and physical activity” with emphasis on four main issues: Why is physical activity important to you? What are your thoughts about physical activity in the years to come? Which factors are important in being able to stay active? What prevents you from being active?

The participants sat around a table and were encouraged to talk and discuss about experiences and situations relating to the themes. Each session lasted 1 hour and included refreshments. In order to enhance the discussion among the participants, a man with experience in conducting focus group interviews, was selected as the moderator. The first author (a woman) who had met the men doing the TGDs, was the assistant and did not participate in the discussions and sat at the back of the room. The co-authors did not participate in the data gathering. This strategy was chosen because we believed that men talk to each other in a different way to how they talk to women. By having a man in the moderator role, we thought the men would talk and discuss more freely. A third FGD was planned, however, when the second came to an end, we experienced little new information beyond the TGD and the first FGD emerged.

### Data analysis

The FGDs were recorded and transcribed verbatim, comprising about 30 pages of written text. In addition, the nine handwritten diaries were included in the analysis. In total this added up to more than 100 pages of written text. Since the aim of this study was to explore challenges and motivators, the qualitative method Systemic Text Condensation [27] was chosen. This is a strategic method in four steps inspired by Giorgi.

#### Step 1

In line with Malterud [27] all text from the FGDs was read several times to get a total impression of the data. The coding process was started by the first author, and discussed further with the co-authors. The emerging themes from each discussion were compared with the themes identified by the first author in written notes after each interview session and between the groups [27]. At this point text from the diaries was added to the analysis process. Each diary text was read and re-read many times in order to get a general understanding of each man’s everyday life.

#### Step 2

Next, the themes were re-read in order to get deeper into the data and sorting meaning units. In line with the analytic process in Systemic text Condensation [27] four codes emerged in the analytic process; exercising, carrying out everyday activities, reasons for being physically active, and advice to other retired men.

#### Step 3

By asking questions, like: What are the men telling us about being physically active? What hindrances to continuing to be physically active have they met? Finally, three categories groups relevant to the research question were identified [27]: retired men and physical activities; perceived obstacles to being able to be physically active; motivation factors in continuing to be physically active.

#### Step 4

**Fig. 1:**
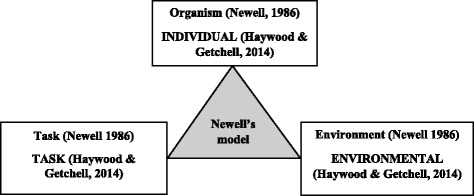
Newell’s schematic diagram of categories of constraint: modified by Haywood and Getchell

After the dynamic analysis process going backwards and forwards, we employed a theoretical model used to study change in movement, to help us get meaning and a deeper understanding of the emerging categories [27]. This model was presented by Newell [28] and has three different categories that interact to determine the optimal pattern of coordination and control for any activity (See Fig. 1).

Haywood and Getchell [29] modified Newell’s model in the book *Life Span Motor Development*, to make sense of developmental changes by providing a framework for observing change. If any of the three categories change, the movement will change. The categories in the model: individual (the person’s unique physical and mental characteristics); environmental (external factors, physical and socio-cultural); and tasks (types of activity).

## Results

**Fig. 2:**
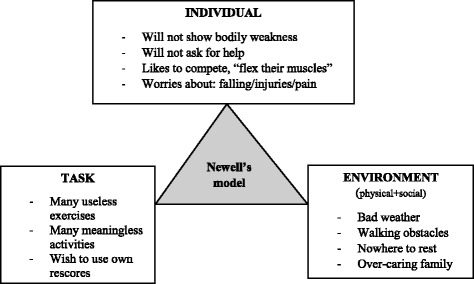
Some perceived challenges for physical activity

The results were organised by using Newell’s diagram, and ended with the following categories: Individual constraints: no weakness, will easily compete and negative thinking; Task constraints: meaningful physical activities; Environmental constraints: physical and social constraints (See Fig. 2).

### Individual constraints: No weakness, will easily compete and negative thinking

The men discussed how gender and their identity as retired men had impacted on physical activity, and pointed out three aspects of this. First, they experienced that they had difficulties showing bodily weakness. “Even a little limp feel like a defect I will not disclose”*.* They confessed that they waited too long before asking for help. This was expressed as: “We have been taught that we men should put up with things more than women and accept our situation, even though we have heard it is important to get early help, like seeing a doctor or a physiotherapist”. The use of assistive technology might also show weakness. For example, when men need walking aids to continue to be active the participants claimed that the majority were reluctant to use them. Interestingly, the men claimed that the use of walking aids was normal for older women, but not for most men. However, men could use Nordic walking poles if necessary. “Poles are associated with sport and with younger people, not with old age”, they told. Even if the men did not use the poles in the way they are meant to be used, they did not feel that their self-esteem and appearance were damaged, unlike with ordinary walking aids. If they really needed a proper walking aid they would rather stay at home than risk meeting people who could observe their weakness.

The second individual aspect was that most men desire to *“*flex their muscles*”*. The participants talked about secretly enjoying doing better than the person next to them. For example, one said that when he started a physical activity, he frequently overdid it to begin with and consequently, had trouble with joints and muscles afterward: “In my mind I am still 30 years old, and when I see others walking fast or working hard I want to be as good, or even better than them”. He compared men and women and said: “Most women are not like that. They are more sensible and start gradually to obtain a higher and higher physical activity level, something we men seem to forget”.

The third individual aspect shows how the men took precautions because of fear of falling or injuring themselves. They had heard and read in the newspapers that injuries will set you back more, and will take longer to heal, than when they were young. “A fall can be the beginning of the end”, one man said. They were terrified of having a fall, therefore they would rather stop walking than risk a fall or injuries. For two or 3 months every year they experienced winterly weather which made walking difficult, and they would be warned by family and friends to be especially careful. Thus, most of them stopped walking in the winter months to make sure they did not fall, and to feel safe from accidents. The consequence was that in spring it was difficult to start walking again, mostly because they were out of shape, but also because of expressions such as this: “I have got out of the habit of walking every day, the walks are not automatic any more”. They all agreed that getting going again after the winter became more and more difficult every year.

The men learnt about risk factors when ageing. This information made them more aware of the risks involved in being physically active, such as falling, and this worried them. They suggested that they needed more advice and help, for example how to stay safe during their daily walks. They would like information about ways to avoid risks rather than always hearing about the possible dangers. They thought perhaps the community could arrange for elderly people to meet and help each other with good ideas. “I have heard some very good ideas today which will give me the courage to walk outside in the winter; like Nordic walking poles and shoes with spikes, and where to buy them”. More positive advice from health professionals or from peers in order to be able to carry on with enjoyable activities are needed.

### Task constraints: Meaningful physical activities

The men knew that physical activity was of great importance when ageing. They read in the newspapers that they should exercise regularly in the gym. Some of them said they knew they needed to make an active and purposeful effort to care for their own health. Many insisted that regular physical activity should be enjoyable and useful. Most of them said they needed a regular programme and good routines to be physical active.

They discussed different types of physical activities. For instance, was it necessary to do exercises or could they be physically active in other ways? For different reasons, more than half the men were responsible for the housework. They were surprised by the effort that went into it, and how tired they were afterwards. One exclaimed: “I had no idea that housework was this physically hard and demanding”*.* Consequently, they felt that it would not be necessary to exercise in the gym on top of doing housework for hours. For an old man, it might even be possible to get help with the housework so that he could go to the gym instead. However, all the men agreed that housework was a good way to combine utility with pleasure, and that this should certainly count as physical activity.

The men wanted to be physically active in their everyday routines, and preferred to be useful and do jobs that needed to be done, like housework, repairs or painting inside or outside the house, and gardening. They could also do physical voluntary work, like taking a person in a wheelchair for a walk or doing repairs and chop wood at the open-air museum. “These types of activities feel more meaningful than exercising in the gym”, one of the men said. Activities like taking care of household tasks made them feel important, which again increased their self-esteem. Friends and family gave them positive feedback and were impressed with these important jobs.

Nevertheless, the men expressed that they needed to be reminded of the value of different types of physical activities. “Whether we like it or not, physical activity is vital when we are ageing, because it is important for our health, and it is up to each one of us to do something about this”. Further, they stated that enjoyable and meaningful activities tailored to individual needs are necessary in order to continue to be active. However, they might need professional help to find good and meaningful activities and to get started with good active habits.

### Environmental constraints: Physical and socio-cultural

Nearly all the men enjoyed walking, and preferred using their own legs for transport as often as possible; for example, walking to meet friends at a coffee shop or getting to the library. In addition, some of them had a regular habit of walking outdoors most days, thus combining being physically active and meeting people. They claimed that when ageing these routines could be difficult to continue because of constraints in the physical environment like poor road surfaces, bad weather or simply needing somewhere to sit down because they did not trust their legs any more. They did not know when their legs would start to hurt, and for that reason some of them had stopped walking in case they needed a rest and were unable to find a suitable place to sit down.

They pointed out that if the politicians and the municipality wanted people to take more responsibility for being physically active, something had to be done in the community. For example, people needed a place to report any obstacles, so that the municipality would be aware of them and then able to fix the problems so that people could continue with their physical routines.

Environmental constraints were also of a social character. Interestingly, the men perceived the influence from people around to have a stronger impact in their old age. “When we are seniors we seem to be more sensitive to how our family and friends think we should behave, I am trusting my own judgement less and less”. The men discussed about family members who stopped them in their physical routines, some of the men felt not trusted. They eventually agreed that this was mostly done by their family to be helpful or caring. However, the result ended up as less physical active. “My children won’t let me walk in the woods alone, as I have always loved to do”. Another talked about his wife looking after him all the time. “When I want to do a little work outside the house, she’s always watching and telling me to be careful”. The men agreed that this made them feel useless and then led to a gradual loss of self-esteem.

They pointed out that people around them were very kind and helpful, but often to such an extent that they were actually prevented from being as physical active as they were capable of and wanted to be, and that this could become an obstacle to activity. One said that when he woke up to a snowfall, his neighbour had already shovelled it away before he even got up. Another talked about his neighbour coming up the stairs every morning with his newspaper: “She is so helpful, however, I needed and would have enjoyed that activity. Now I have no reason to walk down the stairs every morning”.

**Fig. 3:**
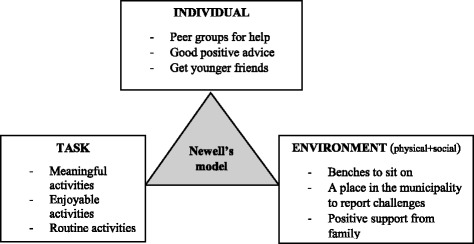
Some perceived motivators for physical activity

As motivators (See Fig. 3) for physical activity, nearly all the men talked enthusiastically about the joy and happiness of spending time with children, mostly grandchildren. Keeping fit to be able to play with them or take them out were important motivators. They also found that they could help them, for example teaching them how to fish, to ski or showing them how to enjoy the natural world. The participants’ well-being and physical fitness were closely linked to mental well-being.

When the men retire, many find new activities to engage in. The focus group participants felt that retirement could be a good time to find a new hobby or an activity that could interest you and could lead to meeting new people, preferably a younger age group. Younger people may make it easier to see new possibilities in life. The participants claimed that. “Many people our own age often are unwell, and talk about illness and problems. Such negative talk can easily make you quite depressed. The younger ones do in contrast talk about other things, such as family, home making and leisure activities”. Finding a hobby or an activity that interests you and can lead to meeting new people, preferably a younger age group, that can make it easier to see new possibilities. Then one could be motivated to continue to be active, both in body and in mind.

## Discussion

### Individual constraints

The men pointed out that since their identity was different to women’s they faced other challenges when ageing. On the one hand, this difference could be positive because they seemed to be more physically active than women [15, 30]. On the other hand, however, we found that when they did have problems with their bodies, they were reluctant to take contact with professionals. In our work with health promotion for the elderly, we have experienced this difference. Men do not like talking about bodily changes or problems, and therefore do not easily ask for help. In addition, since most of the professionals in this field are women, the male perspective appears to be forgotten when designing programmes and giving advice.

In our study, the men claimed that Nordic walking poles give them a more acceptable appearance than other walking aids, because they are associated with young and active people. The device thus speaks an implicit demographic language that feels positive for the men. This illustrates how gender script influences the use or non-use of technology [31]. In addition, results from a systematic review clearly identified that Nordic walking is a healthy and well-accepted mode of physical activity [32], and this support the men’s view of using the poles as a walking aid.

Worrying about falling and having accidents are two-fold. Firstly, preventing falls for the elderly has been a big issue for researchers for many years. This increase in concern has spread as a result of information on television and in the press, therefore, the elderly in the community take precautions and fall less [33]. However, a new issue arising from this seems to be that old people can be too careful because they are afraid, which leads to restriction in activities [17]. The question is therefore whether the second consequence of fall prevention means less physical activity and thus, in the long term, fewer positive health effects. Jefferis et al. [16] argue that fear of falling is an important barrier to gaining health benefits from walking, even more of a barrier than if they have had an actual fall.

Aches and pains in the body seem to be a barrier to physical activity for the men. When starting new activities, they are often vigorous and competitive [21], and start too quickly with too much energy. Consequently, they might experience shortness of breath which leads to worry about the heart, or they get muscle pain and think they are doing wrong activities. They say they need information about their bodies, i.e. what is normal and not dangerous when they start physical activity. However, they had nobody to talk to, and therefore gave up being active, instead of getting help and more knowledge to cope with these barriers. This is in line with Schutzer and Graves [34] reporting that health problems and pain emerged as the most common barrier to being physically active.

### Task constraints

Our study revealed that the men were surprised that everyday activities could be so challenging for the body, both physically for their balance, and for co-ordination. Powell et al. [5] underline this when they discuss physical activity for health. They point out that the value of light activity has just recently being recognised, because current technology has and continues to reduce the need for even light activities in everyday life. When the men had been doing activities like housework and playing with grandchildren, they agreed that then they did not need exercising. It is noteworthy to see that research, from writing about exercising as the only way to be healthy, now points out the importance of other types of physical activity as the men pointed out. Ainsworth et al. [35] have done an important job with Metabolic Equivalents (METS) to be able to compare different types of activities. METS make it possible to see that 30 min of vacuum cleaning is equivalent to 30 min of moderate exercising. The four activity groups [25], show that the elderly men can be sufficiently physically active in their everyday life without doing specific exercises.

Furthermore, the men in this study highlighted the importance of meaningful activities. Baert et al. [36] agree with this, and point out that patients and professions have a different interpretation of what they considered to be physical activity; the patients included physical work and household activities while the professions mainly concentrated on exercises. Further, they [36] stressed the importance of individual programs so that individual barriers can be eliminated and motivators strengthened. Most of the men in our study preferred being useful and feeling needed, rather than putting a lot of effort into useless activities. They wanted to do activities that were important to them individually. Studies support the view that enjoyment and satisfaction give better motivation [8, 34]. Doing voluntary work, it is possible to make use of one’s abilities and practical skills, and in addition it is a way to feel needed and to combine utility with pleasure [37].

Even though the men knew what meaningful activities were for them, they might need a push to get started with new routines. Their GP or other health providers have a key role in helping with more knowledge and effective intervention in order to replace sedentary patterns with habitual physical activity patterns [34].

### Environment

In our study, the seniors wanted an address in the municipality where they could report obstacles. Other studies support this [12] arguing that the built environment can contribute to sedentary behaviour, and that mapping the physical environment in the municipality is important, like safe walking paths and places to rest. Bjornsdottir et al. [38] also found that benches to rest on were much needed. Lately, the removal of physical environmental barriers have been described in many articles, through the provision of good footpaths, benches to rest on and improvement in neighbourhood safety [13, 36, 38]. Takano et al. [39] emphasised that urban planning and political decisions are important to ensure walkable green spaces for senior citizens.

The men in this study revealed that they were more easily influenced by their social environment than before they retired, and Jankowski et al. [40] support this. Studies have pointed out the importance of support from the family to motivate for activities [15, 41, 42]. Surprisingly, we found mostly negative influences from the family as far as physical activities were concerned. When the men in this study talked about being prevented by their family from carrying out some of their important activities, this influence seemed to be a barrier for them. This is supported by Baert et al. [36], who found that spouses were more of a barrier than a motivator to physical activity. Their families were stopping them because they wanted them to avoid injuries. From the men’s point of view, this made them feel old and lacking in confidence. Negative feedback about their capabilities did not facilitate activity. The key here must be more public education about opportunities, rather than risks and dangers, but also about the motivators and what can be done. Family members need to understand their role as motivators [15]. They have identified social support as one of the predictors of being physically active in older populations.

### Strengths and limitations

Even though this was a qualitative study with few participants, we obtained rich and interesting data. The strength is that the participants could comment and give feedback on the summary of the group interviews and of the initial findings given by the first author. Codes and categories were discussed continuously between the three authors until a consensus was reach.

The experiences of the participants are related to them living in Norway, but findings concerning physical activity with challenges and motivators are similar to other studies conducted in countries like USA [12], Iceland [38], Finland [13], Belgium [36] and UK [43].

The findings from this qualitative study have few participants; hence we cannot generalize to other populations. Nevertheless, we assume that these findings can be of interest to professionals working with health programs and health promotion with senior men. More research will be needed to explore how gender differences influence physical activity.

## Conclusion

To be able to achieve the aim of active ageing [7], service providers as well as local authorities need to understand more of the challenges retired men meet when ageing. This pilot study highlights vital aspects of retired men’s challenges in maintaining their physical activity level. It illuminates how retired men’s preferences for physical activity is influenced by gender identities, social roles, physical and social environments, and gives some pointers to the facilitation of physical activity. The findings from this study are important for better understanding men’s challenges when ageing, which is important knowledge for service provision in adjusting programmes to men’s individual preferences. Initiatives should be taken in the municipality to map environmental barriers. The senior citizen need an address to report physical environmental barriers. It is important to implement this knowledge in the practice field, in service delivery and also for people around the men. These findings about gender differences will be taken into consideration in a bigger research program when working in this field. Nevertheless, more research is needed.

## Abbreviations

FGD: Focus group discussion
GP: General practitioner
METS: Metabolic equivalents
NEPA: Non-Exercising Physical Activity
PA: Physical Activity
TGD: Time-Geographic Diary
